# PlantInfoCMS: Scalable Plant Disease Information Collection and Management System for Training AI Models

**DOI:** 10.3390/s23115032

**Published:** 2023-05-24

**Authors:** Dong Jin, Helin Yin, Ri Zheng, Seong Joon Yoo, Yeong Hyeon Gu

**Affiliations:** 1 Department of Computer Science and Engineering, Sejong University, Seoul 05006, Republic of Korea; justdong@sju.ac.kr (D.J.); zrchuangri@sju.ac.kr (R.Z.); sjyoo@sejong.ac.kr (S.J.Y.); 2 Department of Convergence Engineering for Intelligent Drone, Sejong University, Seoul 05006, Republic of Korea; 3 Department of Artificial Intelligence, Sejong University, Seoul 05006, Republic of Korea; yhl0608@sejong.ac.kr

**Keywords:** agricultural dataset, collection and management system, computer vision, deep learning, plant disease and pest detection, PlantInfoCMS

## Abstract

In recent years, the development of deep learning technology has significantly benefited agriculture in domains such as smart and precision farming. Deep learning models require a large amount of high-quality training data. However, collecting and managing large amounts of guaranteed-quality data is a critical issue. To meet these requirements, this study proposes a scalable plant disease information collection and management system (PlantInfoCMS). The proposed PlantInfoCMS consists of data collection, annotation, data inspection, and dashboard modules to generate accurate and high-quality pest and disease image datasets for learning purposes. Additionally, the system provides various statistical functions allowing users to easily check the progress of each task, making management highly efficient. Currently, PlantInfoCMS handles data on 32 types of crops and 185 types of pests and diseases, and stores and manages 301,667 original and 195,124 labeled images. The PlantInfoCMS proposed in this study is expected to significantly contribute to the diagnosis of crop pests and diseases by providing high-quality AI images for learning about and facilitating the management of crop pests and diseases.

## 1. Introduction

Agriculture is one of the oldest and most significant industries in the world. According to the United Nations, the global population is expected to reach approximately 9 billion by 2050 [1], and meeting the increased food demand of the growing population is an important challenge [2]. Therefore, increasing the yield and quality of agricultural products becomes necessary. However, according to the forestry and fishery survey, as of December 2021, the agricultural population in South Korea has decreased by 23.9% compared to the last 10 years, and the aging rate of the agricultural population is 47%. This has led to a chronic shortage of manpower in Korean agriculture, hindering the sustainable development of agriculture. To address these issues, the government is investing heavily in smart farms based on information and communications technology (ICT).

Smart farming incorporates ICT by remotely connecting various information technologies to greenhouses, orchards, livestock barns, etc., and can thus control the growth environment of crops and livestock [3]. Smart farming combines ICT such as remote sensing, Internet of Things (IoT), unmanned aerial vehicles (UAVs), machine learning, artificial intelligence (AI), and networking with traditional agricultural systems such as crop cultivation and livestock farming [4,5]. This enables automatic monitoring and optimization of processes such as environmental conditions, growth status, soil conditions, weed management, and pest and disease management, ultimately improving crop yields and reducing costs [6]. Pest and disease diagnosis and management are major challenges in improving crop yield and quality. Recently, types of pests and diseases have been increasing in number, owing to causes such as trade globalization and climate change [7], thereby increasing farm damages. Therefore, rapid diagnosis and pest and disease control can minimize damage and economic losses on farms.

Numerous fields have effectively utilized the recent advancements in deep learning technology [8], including image-based pest and disease recognition research [9,10]. Building deep learning models requires large amounts of high-quality training data [11]. However, several difficulties exist in constructing training data for crop pests and diseases, as follows:

1. Collecting a large amount of data: It is difficult to collect a large amount of data on crop pests and diseases as they are seasonal [12]. Although data collection is relatively better for pests and diseases that occur frequently, it is extremely difficult in the case of rare, destructive pests and diseases, such as fire blight (*Erwinia amylovora*).
2. Building high-quality data: The key to constructing AI training data is ensuring the quality of the data [13]. An essential task in building AI training data is labeling, which involves marking the data with tags. However, there are many cases where different types of pests and diseases have similar damage symptoms (e.g., fire blight and scab). In such cases, inexperienced individuals may make inaccurate diagnoses, so pest and disease experts need to label the data directly. However, there is a severe shortage of experts [14,15].
3. Consistency of data quality: A comprehensive data management system is needed to ensure effective and consistent data quality [16]. Multiple data sources (e.g., image, location, plant, disease, pesticide info, etc.) are essential to efficiently implement services such as pest and disease occurrence monitoring and image-recognition-based pest and disease diagnosis. This data must be managed in a standardized format using an integrated data management system that can expand the data for long-term use.

To address these issues, an integrated plant disease and pest image management system is needed. Unfortunately, there is currently no system for building and managing image datasets for AI training in the agricultural field. Our research makes the following contributions:

- In this study, we propose a scalable plant disease information collection and management system (PlantInfoCMS) that can efficiently create and manage high-quality image datasets for computer vision tasks.
- Through the app/web-based image collection module, various disease and pest images of crop cultivation sites can be easily collected.
- Through the annotation and inspection module, not only can a training dataset for computer vision in a standardized format be built, but high-quality pest and disease training data can also be stored.
- The dashboard module allows users to check the progress of each task through several statistical functions, and assists administrators in making various decisions based on the information provided.
- Now PlantInfoCMS handles data on 32 types of crops and 185 types of pests and diseases, storing and managing 301,667 original and 195,124 labeled images.

The remainder of the study is organized as follows. Section 2 describes related research, including existing publicly available pest and disease open data and pest and disease data management systems. Section 3 details the proposed method, PlantInfoCMS. Section 4 presents the discussion and limitations. Section 5 concludes the study.

## 2. Related Work

### 2.1. Crop Pest and Disease Datasets

This section describes the publicly available crop pest and disease image datasets. One of the widely used datasets in crop pest and disease recognition research is PlantVillage [17], which consists of 39 categories and 54,303 images, including healthy images. The dataset is built in an ideal laboratory environment, where each image contains a single leaf on a single-color background. Only image-level annotations are provided, making it suitable for classification purposes. However, laboratory environments can differ significantly from actual cultivation environments and the recognition performance of models trained with laboratory-built datasets may significantly deteriorate when applied to real-world environments [18].

The IP102 dataset [19] is a large-scale pest dataset consisting of 75,000 images and 102 categories. In this dataset, most images only provide image-level annotations for training classification models, and only 1900 images provide instance-level annotations that can be used for training object detection models.

AIHub [20], an AI training data platform, provides large-scale pest and disease datasets, such as the fire blight shooting image dataset [21], open-field crop disease diagnosis image dataset [22], and open-field crop pest diagnosis image dataset [23]. The fire blight shooting image dataset [21] contains 9 classes and 211,555 images, and among the photographed images, 95,000 are normal images and 15,000 are disease images. Most images are normal images without diseases, and the disease images are generated through data augmentation techniques such as rotation and flipping. The open-field crop disease diagnosis image dataset [22] provides disease data for 10 types of open-field crops for disease diagnosis. This dataset contains 21 pest and disease classes and 349,151 images, but only 30,000 of them are original images, the rest are normal images or images obtained through data augmentation. The open-field crop pest diagnosis image dataset [23] provides pest image data of the 10 major open-field crops for the development of pest and pest damage diagnosis models and includes 20 classes and 502,530 images. Although AIHub provides large-scale pest and disease image datasets [21,22,23], most of them are normal images without diseases or pests, or images obtained through data augmentation, with only a small proportion of pest and disease images. Many long-distance shots and out-of-focus images are inaccurate for training data.

AgriPest [24] is a domain-specific benchmark dataset for tiny wild pest recognition and detection. AgriPest captures 49.7K images of 4 crops containing 14 species of pests in the field environment. However, this dataset is limited to the pest field, containing only tiny wild pests, which restricts its applicability.

Table 1 presents a summary of datasets for crop pest and disease images that were introduced earlier. However, there are several limitations with the existing datasets for training various pest and disease diagnosis models.

For instance, the PlantVillage dataset, constructed in a laboratory setting, exhibits significant differences from datasets of images captured in real-world agricultural settings, potentially leading to reduced performance of models trained on the PlantVillage dataset when deployed in the field. Furthermore, since only image-level annotations are provided, the dataset can only be used for training disease classification models. While the IP102 and AgriPest datasets provide annotations for training detection models, they are limited in the number and variety of images. Additionally, both datasets only cover pest images. There are few large datasets available for training detection models due to the lack of a system for collecting and managing crop pest images on a large scale and the high cost of constructing datasets. The open datasets related to pest and disease images provided by AIHub comprise hundreds of thousands of images; however, over half of them feature normal crop images without pests and diseases. Furthermore, there are errors in some annotations and inconsistencies in the annotation levels, making it challenging to use for training detection models. Therefore, careful inspection of images and annotations is crucial when collecting pest images, and this issue has yet to be resolved. To address the shortage of large-scale pest image datasets, an efficient system is needed for collecting and managing pest images, conducting inspections on images and annotations.

### 2.2. Pest and Disease Image Data Management System

Various domains have developed data collection and management systems to efficiently manage AI training datasets. Particularly, the medical field is actively developing systems to manage medical data such as CT and MRI scans [25,26]. In the agricultural field, most data management systems primarily focus on crop growth monitoring [27], plant phenotyping data [28,29], geo-referenced data [30], and breeding data management [31].

A study by [27] proposed a crop disease management (CDM) system using IoT technology. The CDM system monitors crops with charge-coupled device cameras and identifies diseases using various statistical methods in order to recommend appropriate pesticides to farmers. Although CDM deals with crop images, the system focuses on disease monitoring rather than image management. As such, it is of limited use in image analysis and management.

CropSight [29] is a server platform based on PHP Hypertext Preprocessor and structured query language, which provides automated data collection, storage, and information management functions through distributed IoT centers and phenotyping workstations. CropSight can be used to maintain and collect crop performance and microclimate datasets captured by IoT sensors and distributed phenotyping installations. Although CropSight offers various analysis methods, most target phenotyping and microclimate data, making it inappropriate to be used as an efficient image data management system.

IAP [28] is an open-source framework for high-throughput plant phenotyping that applies image processing techniques to quantify plant growth and performance based on phenotypic traits. IAP provides various functions for import and export management and analyzing plant phenotyping data via a user-friendly interface. The validity of the proposed platform has been proven using maize plants grown in greenhouse environments. Although IAP uses plant images for plant phenotyping, it has limitations in managing images and performing statistical analyses.

Yang et al. [30] proposed a cropland and soil data management system that enables automatic data consolidation and integration, and provides dynamic access to integrated data. The system integrates data from agricultural land for crops such as rice, corn, cotton, alfalfa, and sorghum, as well as non-agricultural land data such as woodland, shrubland, and urban areas, and visualizes them using a map viewer.

Jung et al. [31] proposed a breeding information management system (BIMS) that efficiently manages crop breeding data. BIMS offers features that allow breeders to store, manage, archive, and save breeding data, and provides functions to analyze and archive integrated data from both private phenotypic and genotypic data owned by individual breeders and publicly available data through a web-based interface.

Table 2 summarizes the modality, domains, and other characteristics of the data handled by the systems proposed in each of the studies introduced earlier. Yang et al.’s and BIMS’s systems focus on managing structured tabular data for cropland and breeding data management, and it is impossible to efficiently collect and manage images. CropSight and IAP are both capable of efficiently managing large-scale image data. However, their primary focus lies in image analysis, specifically aiding in plant phenotyping tasks. Furthermore, since most crop images collected for plant phenotyping do not include diseases or pests, these systems are not suitable for building datasets for training crop pest detection models. CDM is a system focused on disease monitoring, and uses charge-coupled device cameras to diagnose crop disease from images, rather than collecting disease images. As such, existing studies have focused on managing and monitoring crop images. To construct high-quality datasets that are suitable for model training, it is essential for systems to offer support for image annotation and inspection. However, existing systems do not provide such functionalities. The research on systems specifically designed for collecting images to train crop pest and disease detection models and creating large-scale training datasets is scarce. To collect a substantial number of images simultaneously, multiple edge device users must be able to collect and upload data simultaneously. However, existing systems do not support this functionality, making it difficult to collect a large amount of various crop pest and disease images in a short amount of time.

The various image monitoring and analysis tools proposed in this study are designed for efficient image collection and data construction. Through a smartphone application, multiple users can collect data simultaneously, enabling the construction of large-scale datasets. The system was implemented with a scalable design to easily expand the types of crops, pests and disease images that can be collected. Furthermore, an image and annotation inspection process involving pest and disease experts are introduced to ensure the construction of more reliable datasets.

## 3. PlantInfoCMS

As revealed through related studies, existing pest and disease image management systems do not prioritize data construction and inspection functions to create reliable datasets. Furthermore, due to the lack of suitable tools, there is also a shortage of available large-scale, high-quality pest and disease image datasets for AI training. In this study, we propose PlantInfoCMS to address these issues by introducing efficient image collection, management, and inspection capabilities. Our proposed system allows multiple users to simultaneously collect and upload images via a mobile application, and facilitates efficient management through various visualization methods. Additionally, an inspection process is introduced to ensure the quality of the collected images.

**Figure 1:**
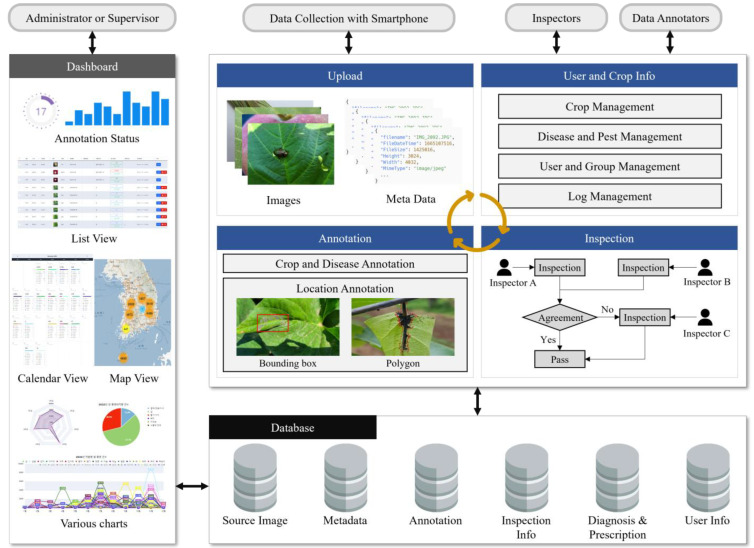
Diagram of PlantInfoCMS. PlantInfoCMS is comprised of modules for image upload, image annotation, image inspection, user and crop information management, and a dashboard.

The PlantInfoCMS proposed in this study includes upload, annotation, inspection, user and crop info, and dashboard modules as shown in Figure 1. In the upload module, users input the crop pest and disease images, and the annotation module specifies the damaged areas in the original images to create AI training data. In the inspection module, the uploaded original images or annotated images are reviewed to ensure accurate and high-quality training images. The user and crop info management module manages user, crop, pest, and disease information. Finally, the dashboard module displays the progress of tasks such as image uploads, annotations, and schedules using various visualization tools.

The proposed system can be described as a multi-tiered architecture from a software engineering perspective. At the highest level, the system has a front-end interface that enables users to upload images and manage them based on crop, disease, or pest level. The interface is implemented as a web-based application using the jQuery and Bootstrap libraries. The middle level of the system consists of the image management, annotation, and inspection module, which provides features such as image upload, annotation progress status analysis, and image inspection logic. This module is responsible for managing and processing the large volume of images collected by the system, and is implemented using Hypertext Preprocessor (PHP). At the lowest tier, the system includes an image storage system, which is responsible for storing the images and associated metadata such as crop type, disease type, and annotation information. The MySQL database is employed to store the images and metadata. Each module will be explained in detail in the sections below.

**Figure 2:**
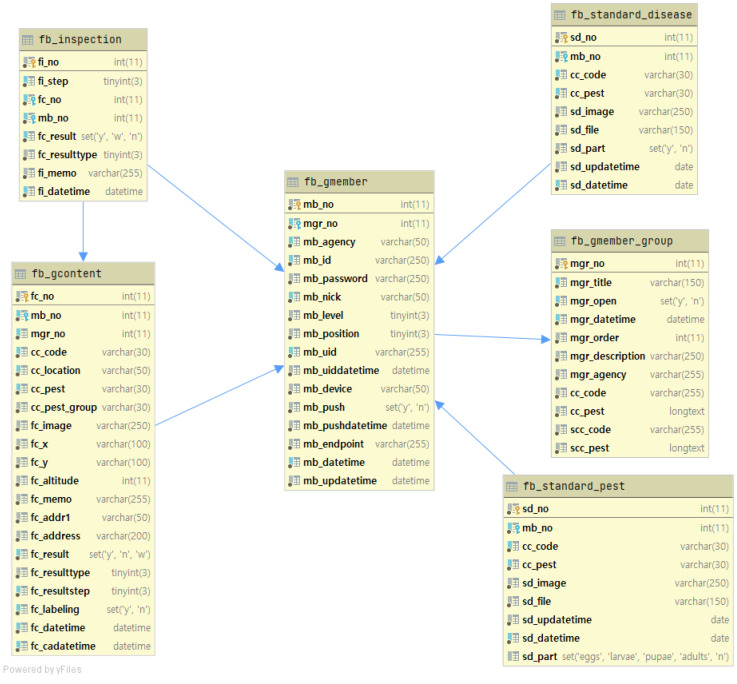
Entity relationship diagram of PlantInfoCMS. The ERD illustrates the database structure design for the primary tables employed in the system. The tables responsible for storing uploaded images, user information, and image inspection data are interconnected using foreign keys.

Figure 2 shows the design of the database structure for the main tables used in the system. The fb_standard_pest and fb_standard_disease tables in the database store information about uploaded pest and disease images, respectively. The fb_gmember table stores users’ information, while the fb_gcontent table stores metadata related to the images uploaded by users. The metadata includes information such as the crop type, pest or disease type, and shooting location, and is linked to the table that stores information about the uploaded images using foreign keys. The fb_inspection table is used to record the results of pest and disease expert inspection for each image. This table is connected to the fb_gcontent and fb_gmember tables using foreign keys to manage the metadata of the images to be inspected and the information of the users who uploaded the images.

### 3.1. Pest and Disease Image Upload Module

The pest and disease image upload module receives crop pest and disease images taken by users and uploads them to the server. The users are crop pest and disease experts who are affiliated with the government agency, the Rural Development Administration (RDA). These users gather several pest and disease images taken in the field through regular site inspections and communication with local farmers.

**Figure 3:**
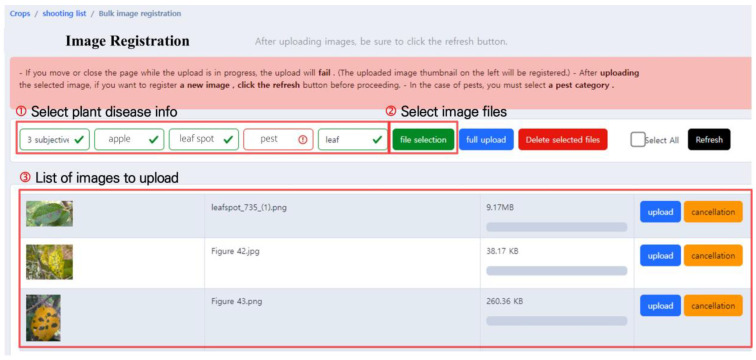
Pest and disease image upload screen. This user interface allows users to conveniently upload one or multiple images simultaneously.

The pest and disease image upload screen is shown in Figure 3. Users should first select the crop pest and disease information for the image to be uploaded, as shown in Figure 3. ➀, and select the affiliation of the uploader, crop type, pest, or disease type, form of the pest (only applicable for pests), and the affected area. Seven uploader affiliations are currently defined, and each affiliation handles different types of crops, pests, and diseases. There are 32 types of crops, such as apples and pears, and users can select pests and diseases that occur in the chosen crop from a given list. Users can only select the pest from the pest category in the previous pest and disease selection step, and there are four categories to select from: egg, larva, adult, and damage caused by pests. Finally, when selecting the affected area, users can specify the damaged part of the crop, such as leaves, fruit, stems, or roots.

After selecting the crop pest and disease information, the “Select image files” button in Figure 3. ➁ is used to select the images to be uploaded, and multiple images can be selected simultaneously. Only JPG and PNG formats are supported. The selected images are then displayed on the screen as shown in Figure 3. ➂, and users can either upload the images or cancel the upload after rechecking.

After the crop pest and disease image upload process, information such as the person who uploaded the image, pest or disease, and image gets automatically stored in the database; the items are summarized in Table 3. Most items in Table 3 are generated automatically; however, crop names, pest and disease names, pest forms, and affected areas of the uploaded images should be manually selected through the selection menu in Figure 3. ➀. The system is designed to automatically parse information about the captured images from its metadata. For example, “shooting location information” is automatically stored in the metadata when the GPS function is enabled on the shooting device, but it is not stored if GPS is not enabled. As location information can be useful for data management and visualization, we have designed the system such that users manually add it when the location information is not provided. Currently, image location information is used only for visualizing the map view. In the future, it can be utilized to analyze the distribution of crops nationwide and patterns of pest occurrence. Section 3.4 will provide a detailed introduction to the visualization method of map view which utilizes location information.

**Figure 4:**
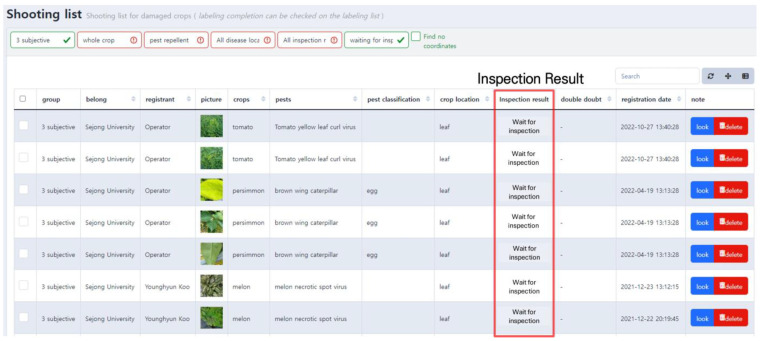
Screen of uploaded pest and disease image list. This user interface enables users to conveniently access information including the uploader’s name, crop name, pest and disease names, and inspection results.

The uploaded pest and disease images and other information are displayed as shown in Figure 4. The person who uploaded the image can reconfirm the information on this screen and make any corrections. Uploaded images are marked as “waiting for inspection,” and they eventually become training data after going through the annotation and inspection modules.

### 3.2. Pest and Disease Image Annotation Module

In the pest and disease image annotation module, labeling is performed to mark the affected areas in the images. Labeling is a crucial step in building training data for AI, as the labeled data are used to train AI models.

**Figure 5:**
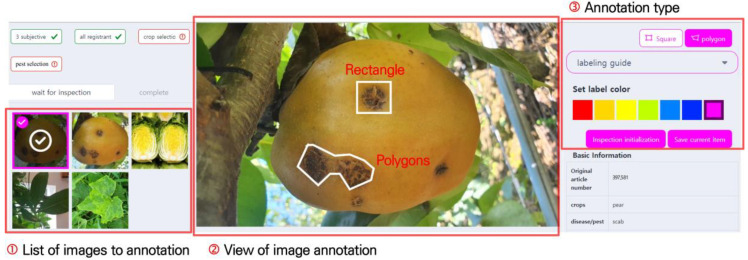
Image annotation screen. Users can select images for annotation from the ➀ list of images to annotate. The system supports two annotation methods, as indicated by the ➂ annotation type: rectangles and polygons.

The pest and disease image annotation screen is shown in Figure 5. Figure 5. ➀ is the annotation image waiting list, which displays the uploaded images. Upon clicking an image, it is enlarged and displayed as shown in Figure 5. ➁, and the affected areas can be marked. For annotation, rectangles and polygons are supported. Images marked with rectangles can be used in object detection tasks, and the annotation information contains coordinate values in the format ($X_{1},X_{2},Y_{1},Y_{2}$). As pest and disease symptoms have various forms, there are limitations to marking them with rectangles. To solve this, a feature was added to allow marking in polygon format, which enabled users to mark complex damage symptoms more precisely. Images marked with polygons can be used in object detection and segmentation tasks. The annotation information is automatically saved in a separate JSON file.

### 3.3. Pest and Disease Image Inspection Module

The quality of labeled images is inspected in the pest and disease image inspection module.

Image inspections are performed by pest and disease experts, and only those who have been pre-approved gain access to the inspection module. PlantInfoCMS allows only users of a specific group authorized by the administrator through the group management feature to visit the module. Pest and disease experts inspect the annotated images for the following:

(1) Check whether the captured images include diseases or pests and whether the original image is too dark or over-exposed.
(2) Verify whether the captured image is assigned the correct crop name and pest or disease name.
(3) Inspect whether the affected areas of disease symptoms or pest occurrence have been accurately annotated without omissions.

**Figure 6:**
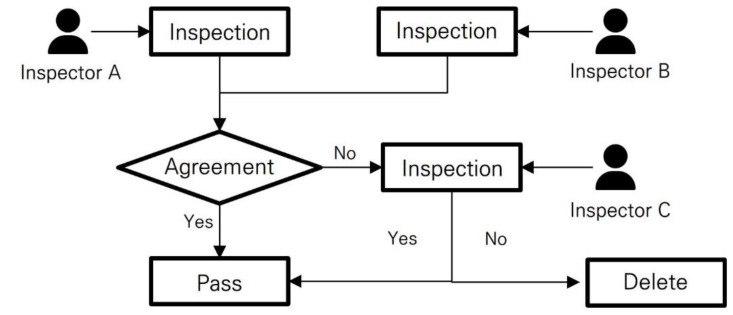
Flow chart describing image inspection process. This flowchart illustrates the step-by-step procedure followed by crop pest and disease experts during the inspection of a single image.

The inspection module is designed to have at least two inspectors review a single image. The inspection process for one image is as follows (also shown in Figure 6):

(1) Two inspectors each review a single image and assign a status of “inspection passed” or “inspection not passed”.
(2) If both inspectors agree, then their opinion (passed or not passed) is used as the final inspection result.
(3) If the two inspectors have different opinions, then a third inspector determines the final inspection result.

Information about these inspections is stored in the inspection database, and if an image fails the inspection, feedback is provided to the person who annotated the image through a notification to make corrections.

### 3.4. Dashboard Module

**Figure 7:**
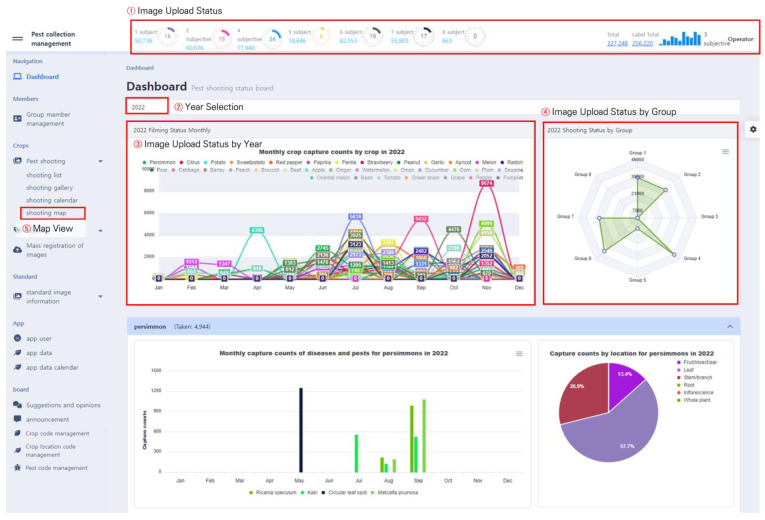
Dashboard main screen. The dashboard provides various visualization graphs, such as status bars, pie charts, and radar charts, to facilitate monitoring the progress of image uploads and annotation tasks.

PlantInfoCMS provides various visualization graphs through the dashboard, making it easy to check the progress of image uploads and annotation tasks. The users can check not only the upload and annotation progress of each group by month, crop, and group, but can also check on the task progress by date and shooting location through visualization methods such as calendar view and map view. The dashboard main screen displayed to users in PlantInfoCMS is shown in Figure 7.

**Figure 8:**
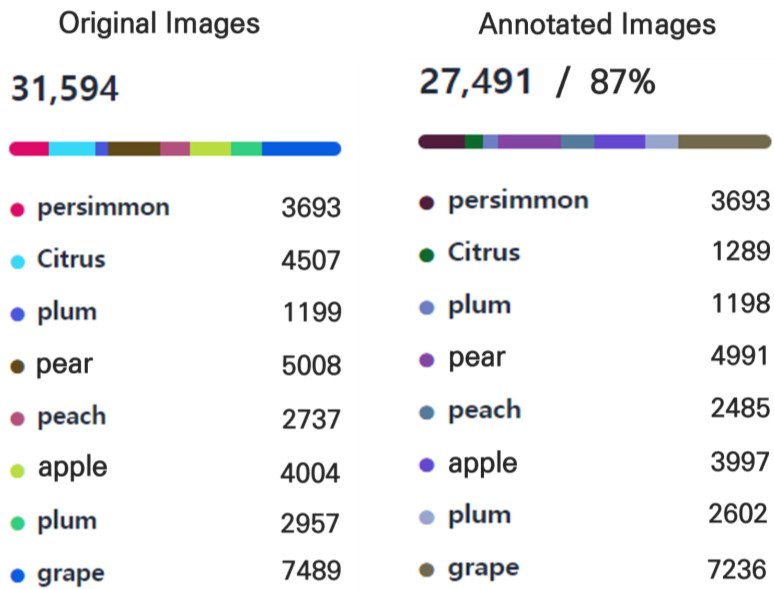
Status of image upload/annotation tasks by group. This panel enables users to conveniently track the status of image uploads and annotation tasks categorized by groups.

It consists of a status bar, menu bar, and panels that display various visualization results. As shown in Figure 7. ①, the Image Upload Status provides features to check the image upload and annotation status of each group through pie charts and bar charts. The pie chart displays the number of uploaded images in each group, the number of annotated images, and the proportion of annotated images among the total images. Upon clicking the pie chart, the image upload and annotation task status for each group are displayed as percentages, as shown in Figure 8, allowing users to briefly check the progress.

Using the Year Selection feature in Figure 7. ②, users can select a specific year and view the corresponding task statistics. The feature Image Upload Status by Year in Figure 7. ③ visualizes the number of uploaded pest and disease images using a line graph and it shows the number of image uploads for each crop per month. The status of images uploaded in each group is displayed in Figure 7. ④ using a radar chart, making it convenient to check the group with the most or least uploaded images.

**Figure 9:**
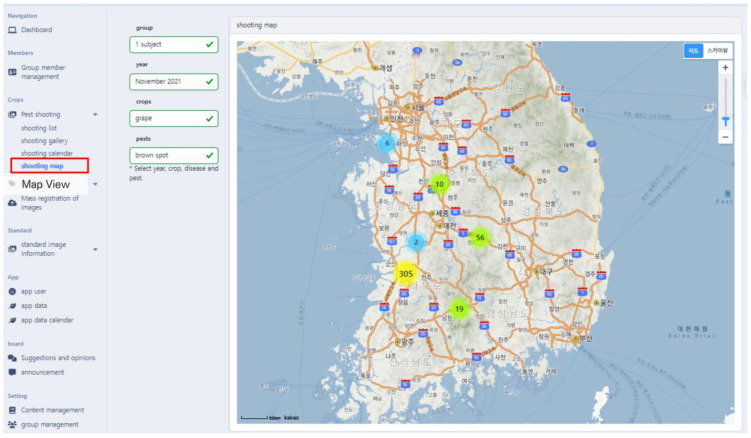
Map view screen. The map view feature enables users to check the status of pest and disease image uploads by region, as well as visualize the geographical distribution of uploaded images. The Korean text on the map indicates the names of cities and bodies of water.

PlantInfoCMS supports a map view that allows the user to check the pest and disease image upload status by region. Map View, featured in Figure 7. ⑤, allows users to easily identify the shooting location and distribution of the uploaded images. The map view, as shown in Figure 9, was implemented using the metadata of the image (e.g., location). The shooting location metadata of the image is stored to one day expand the system into a control system in the future.

## 4. Discussion and Limitations

This study introduces PlantInfoCMS, a scalable system designed for the efficient collection and management of large-scale crop pest and disease data. The proposed system consists of data upload, annotation, inspection, and dashboard modules. The data upload module provides plant pest and disease experts with a user-friendly interface and automatic parsing of image metadata, simplifying the process of uploading images taken in the field. The annotation module offers labeling functions using rectangles and polygons, making it easy to create datasets for object detection and segmentation model training. In the image inspection module, we introduced a process where at least two pest and diseases experts inspect each image to ensure the quality of the collected data. The dashboard provides various charts and a map view for easily visualizing the shooting locations and distribution of the uploaded images.

Previous research on data collection and management systems in the agricultural field has primarily focused on managing tabular data such as plant phenotyping or breeding data, with limited research on systems for managing image data. This study aims to address this issue by proposing a system that efficiently collects and manages plant pest and disease images and builds datasets. It is expected that the proposed system will facilitate the creation of multiple pest image datasets. Currently, PlantInfoCMS has collected a total of 301,667 images, including 185 pest and disease images from 32 different crops.

However, the current system has limitations in that it only manages image data. Future research aims to integrate the system with the open API provided by the Korea Meteorological Administration to enable the analysis and management of weather and climate information at the image-capturing location at the time of image capture. Additionally, the system plans to add the functionality to analyze data from soil and temperature sensors used in smart farms and integrate with the National Crop Pest Management System (NCPMS) of the Rural Development Administration (RDA) of Korea to enable diagnosis and treatment prescription information on the pests and diseases to be checked within the system.

The currently developed system for collecting and managing images of pests and diseases distinguishes images by crop, disease, and pest levels. Crop images can be divided into leaves, stems, branches, fruits, etc. depending on the shooting position, while diseases can be divided into early stage, mid-stage, and late stage according to the onset time. Pests can also be divided into eggs, larvae, pupae, adults, etc. depending on their growth stage. Incorporating such detailed information in the annotation during dataset construction enables more sophisticated diagnosis of diseases and pests, and can serve as evidence for assessing disease severity. Therefore, in future research, we plan to expand the system to include more detailed information such as the parts of the crop image captured, the progression of diseases in terms of early, mid, and late stages, and the growth stages of pests, in the dataset construction.

The currently developed plant disease information collection and management system focuses on pest and disease image collection and dataset construction. However, early diagnosis of crop pests and diseases cannot be adequately resolved using only simple vision data. To achieve more accurate early diagnosis of plant pests and diseases, crop growth data and weather data must also be utilized. For instance, a more accurate diagnosis can be obtained by comprehensively analyzing the diagnostic results of image-based prediction models and the growth information of the corresponding crops when diagnosing a crop disease. By using growth meta-information to determine whether the disease predicted by the model is likely to occur at the time of prediction in an actual farm, comprehensive diagnosis results can be provided to the user. Nevertheless, the current system only focuses on pest and disease image collection and dataset construction and lacks the functionality to collect, manage, and analyze data related to crop production, weather information, and other data. In future research, we plan to expand PlantInfoCMS to include the ability to collect, manage, and analyze data related to crop production, such as growth data and weather data.

## 5. Conclusions

This study proposed PlantInfoCMS, a system for efficiently collecting and managing crop pest and disease image data. The proposed system consists of data collection, annotation, inspection, and dashboard modules to create high-quality pest and disease training data through these processes. Moreover, the proposed system has excellent scalability and provides various statistical functions, making management highly efficient by allowing users to easily check the progress of each task.

In this study, we propose a system for collecting crop pest and disease images and constructing a high-quality dataset. With the proposed system, we collected approximately 301,557 pest and disease images and conducted annotation and inspection for 255,183 of those images. However, we have not yet systematically validated the accuracy of the diagnostic model trained using the dataset constructed with the system. In future research, we plan to train a pest and disease diagnostic model using the constructed dataset, and test the feasibility of using the dataset through accuracy verification.

## Figures and Tables

**Table 1:** Summary of crop pest and disease datasets. The tasks that can be trained using each dataset, the number of classes, and the size of the dataset were analyzed. The number of classes indicates the total number of classes present in the dataset, while the size refers to the total number of images in the dataset.

Dataset	Task	Number of Classes	Size
PlantVillage [17]	Classification	38	54,303
IP102 [19]	Classification, Detection	102	75,222
AgriPest [24]	Detection	14	49K
Fire blight shooting image dataset [21]	Detection	9	211,555
Open-field crop disease diagnosis image dataset [22]	Detection	21	349,151
Open-field crop pest diagnosis image dataset [23]	Detection	20	502,530

**Table 2:** Summary of the system for crop-related data management. The summary is based on the domain and modality of the data being handled by the system, as well as whether the system supports image annotation and image inspection.

Study	Domain	Modality	Task	Image Annotation	Image Inspection	Scalability
CDM System [27]	Crop disease	Image, Tabular	Crop disease monitoring	X	X	X
CropSight [29]	Plant phenotyping	Image, Tabular	Plant phenotyping, environmental and crop growth monitoring	X	X	O
IAP [28]	Plant phenotyping	Image, Tabular	Plant phenotyping	X	X	O
Yang et al. [30]	Cropland and soil	Tabular	Crop production management and analysis	X	X	O
BIMS [31]	Crop breeding	Tabular	Breeding information management	X	X	O
PlantInfoCMS(ours)	Crop disease and pest	Image	Building a dataset for image-based pest and disease diagnosis.	O	O	O

**Table 3:** Image File Metadata Standardization Table. The table stores information about the users who uploaded the images, information about the crops, pests, and disease types included in the images, as well as location information about where the images were obtained.

Item	Column Name	Description	Manual/Automatic Generation
Affiliation Name	AUTHOR_GROUP_NM	Example: 3rd group	Automatic
Uploader Name	UPLOADER_NM	Person who uploaded the image	Automatic
Crop Name	CROPS_NM	Example: apple, pear	Manual (selection window provided)
Disease/Pest Name	DBYHS_NM	Example: fire blight	Manual (selection window provided)
Pest Form	HI_CL	Example: egg, larva, adult	Manual (selection window provided)
Affected Area	POTOGRF_REGN_NM	Example: leaf, stem, flower	Manual (selection window provided)
Shooting Location Information	POTOGRF_PLACE_INFO	Shooting Location Address	Automatic/Manual
Shooting Date	DTA_REGIST_DATETM	Example: 2020.11.20. 17:08:15	Automatic
Labeling Date	DTA_LBL_REGIST_DATETM	Date when the image was uploaded	Automatic
Latitude	POTOGRF_YCRD	Latitude of shooting location	Automatic
longitude	POTOGRF_XCRD	Longitude of shooting location	Automatic
Image File Name	IMAGE_FILE_NM	Example: DSC_0001.jpg	Automatic
Image File ID	IMAGE_FILE_ID	Example: 0000001	Automatic
Image URL	IMAGE_URL	Image URL for external calls	Automatic
Image Path	FILE_STRE_COURS	Image storage location for internal calls	Automatic
Image size	IMAGE_SIZE	Image length and width example: DLALWL ZMRL, 4031*1080	Automatic
Resolution	RESOLUTION_INFO	Example: FHD (1920*1080)	Automatic
Device Information	CMRA_INFO	Smartphone Device Information	Automatic

## Data Availability

Not applicable.

## References

[1] UN United Nations Population Division. https://www.un.org/development/desa/pd/.

[2] Araus J.L., Cairns J.E. (2014). Field high-throughput phenotyping: The new crop breeding frontier. Trends Plant Sci..

[3] Yoon C., Lim D., Park C. (2020). Factors affecting adoption of smart farms: The case of Korea. Comput. Human Behav..

[4] Triantafyllou A., Tsouros D.C., Sarigiannidis P., Bibi S. An architecture model for smart farming. Proceedings of the 2019 15th International Conference on Distributed Computing in Sensor Systems (DCOSS).

[5] Sundmaeker H., Verdouw C., Wolfert S., Freire L.P. (2022). Internet of Food and Farm 2020. Internet of Things Connecting the Physical, Digital and Virtual Worlds.

[6] Boursianis A.D., Papadopoulou M.S., Diamantoulakis P., Liopa-Tsakalidi A., Barouchas P., Salahas G., Karagiannidis G., Wan S., Goudos S.K. (2022). Internet of Things (IoT) and Agricultural Unmanned Aerial Vehicles (UAVs) in smart farming: A comprehensive review. Internet Things.

[7] Aji G.K., Hatou K., Morimoto T. (2020). Modeling the dynamic response of plant growth to root zone temperature in hydroponic chili pepper plant using neural networks. Agriculture.

[8] Yu H., Miao C., Leung C., White T.J. (2017). Towards AI-powered personalization in MOOC learning. NPJ Sci. Learn..

[9] Dhaka V.S., Meena S.V., Rani G., Sinwar D., Kavita, Ijaz M.F., Woźniak M. (2021). A survey of deep convolutional neural networks applied for prediction of plant leaf diseases. Sensors.

[10] Atila Ü., Uçar M., Akyol K., Uçar E. (2021). Plant leaf disease classification using EfficientNet deep learning model. Ecol. Inform..

[11] Kaya A., Keceli A.S., Catal C., Yalic H.Y., Temucin H., Tekinerdogan B. (2019). Analysis of transfer learning for deep neural network based plant classification models. Comput. Electron. Agric..

[12] Liu J., Wang X. (2021). Plant diseases and pests detection based on deep learning: A review. Plant Methods.

[13] Saiz-Rubio V., Rovira-Más F. (2020). From smart farming towards agriculture 5.0: A review on crop data management. Agronomy.

[14] Yin H., Gu Y.H., Park C.J., Park J.H., Yoo S.J. (2020). Transfer learning-based search model for hot pepper diseases and pests. Agriculture.

[15] Gu Y.H., Yin H., Jin D., Zheng R., Yoo S.J. (2022). Improved Multi-Plant Disease Recognition Method Using Deep Convolutional Neural Networks in Six Diseases of Apples and Pears. Agriculture.

[16] Wong Z.S.Y., Zhou J., Zhang Q. (2019). Artificial Intelligence for infectious disease Big Data Analytics. Infect. Dis. Health.

[17] Hughes D.P., Salathe M. (2015). An open access repository of images on plant health to enable the development of mobile disease diagnostics. arXiv.

[18] Cap Q.H., Uga H., Kagiwada S., Iyatomi H. (2022). LeafGAN: An Effective Data Augmentation Method for Practical Plant Disease Diagnosis. IEEE Trans. Autom. Sci. Eng..

[19] Wu X., Zhan C., Lai Y.K., Cheng M.M., Yang J. IP102: A Large-Scale Benchmark Dataset for Insect Pest Recognition. Proceedings of the IEEE/CVF Conference on Computer Vision and Pattern Recognition (CVPR).

[20] AIHUB An AI Training Data Platform. https://aihub.or.kr/.

[21] Fruit Fire Blight Shooting Image Dataset. https://aihub.or.kr/aihubdata/data/view.do?currMenu=115&topMenu=100&aihubDataSe=realm&dataSetSn=146.

[22] The Open-Field Crop Disease Diagnosis Image Dataset. https://aihub.or.kr/aihubdata/data/view.do?currMenu=115&topMenu=100&aihubDataSe=realm&dataSetSn=147.

[23] The Open-Field Crop Pest Diagnosis Image Dataset. https://aihub.or.kr/aihubdata/data/view.do?currMenu=115&topMenu=100&aihubDataSe=realm&dataSetSn=148.

[24] Wang R., Liu L., Xie C., Yang P., Li R., Zhou M. (2021). Agripest: A large-scale domain-specific benchmark dataset for practical agricultural pest detection in the wild. Sensors.

[25] Patel V. (2019). A framework for secure and decentralized sharing of medical imaging data via blockchain consensus. Health Inform. J..

[26] Tournier J.D., Smith R., Raffelt D., Tabbara R., Dhollander T., Pietsch M., Christiaens D., Jeurissen B., Yeh C.H., Connelly A. (2019). MRtrix3: A fast, flexible and open software framework for medical image processing and visualisation. Neuroimage.

[27] Seetharaman K. (2021). A Fully Automated Crop Disease Monitoring and Management System Based on IoT: IoT-Based Disease Identification for Banana Leaf. Deep Learning Applications and Intelligent Decision Making in Engineering.

[28] Klukas C., Chen D., Pape J.M. (2014). Integrated analysis platform: An open-source information system for high-throughput plant phenotyping. Plant Physiol..

[29] Reynolds D., Ball J., Bauer A., Davey R., Griffiths S., Zhou J. (2019). CropSight: A scalable and open-source information management system for distributed plant phenotyping and IoT-based crop management. Gigascience.

[30] Yang Y., Wilson L.T., Wang J., Li X. (2011). Development of an integrated Cropland and Soil Data Management system for cropping system applications. Comput. Electron. Agric..

[31] Jung S., Lee T., Gasic K., Campbell B.T., Yu J., Humann J., Ru S., Edge-Garza D., Hough H., Main D. (2021). The Breeding Information Management System (BIMS): An online resource for crop breeding. Database.

